# First cases of SARS-CoV-2 BA.2.86 in Denmark, 2023

**DOI:** 10.2807/1560-7917.ES.2023.28.36.2300460

**Published:** 2023-09-07

**Authors:** Morten Rasmussen, Frederik Trier Møller, Vithiagaran Gunalan, Sharmin Baig, Marc Bennedbæk, Lasse Engbo Christiansen, Arieh Sierra Cohen, Kirsten Ellegaard, Anders Fomsgaard, Kristina Træholt Franck, Nicolai Balle Larsen, Tine Graakjær Larsen, Ria Lassaunière, Charlotta Polacek, Amanda Gammelby Qvesel, Raphael Niklaus Sieber, Lasse Dam Rasmussen, Marc Stegger, Katja Spiess, Man-Hung Eric Tang, Lasse Skafte Vestergaard, Thomas Emil Andersen, Silje Vermedal Hoegh, Rune Micha Pedersen, Marianne Nielsine Skov, Kat Steinke, Thomas Vognbjerg Sydenham, Morten Hoppe, Lene Nielsen, Tyra Grove Krause, Henrik Ullum, Pikka Jokelainen

**Affiliations:** 1 Virus Research and Development Laboratory, Virus & Microbiological Special Diagnostics, Statens Serum Institut, Copenhagen, Denmark; 2 Infectious Disease Epidemiology and Prevention, Statens Serum Institut, Copenhagen, Denmark; 3 Sequencing and Bioinformatics, Bacteria, Parasites & Fungi, Statens Serum Institut, Copenhagen, Denmark; 4 Epidemiology Research, Statens Serum Institut, Copenhagen, Denmark; 5 TestCenter Denmark, Statens Serum Institut, Copenhagen, Denmark; 6 Virus Surveillance and Research Laboratory, Virus & Microbiological Special Diagnostics, Statens Serum Institut, Copenhagen, Denmark; 7 Antimicrobial Resistance and Infectious Diseases Laboratory, Harry Butler Institute, Murdoch University, Perth, Australia; 8 Department of Clinical Microbiology, Odense University Hospital, Odense, Denmark; 9 Research Unit for Clinical Microbiology, University of Southern Denmark, Odense, Denmark; 10 Department of Clinical Microbiology, Copenhagen University Hospital, Herlev and Gentofte, Denmark; 11 Epidemiological Infectious Disease Preparedness, Statens Serum Institut, Copenhagen, Denmark; 12 Statens Serum Institut, Copenhagen, Denmark; 13 Infectious Disease Preparedness, Statens Serum Institut, Copenhagen, Denmark; * These authors contributed equally to this work and share first authorship.

**Keywords:** COVID-19, emerging variant, diagnostic preparedness, epidemiological preparedness, SARS-CoV-2, sequencing, wastewater surveillance, virus, WGS

## Abstract

We describe 10 cases of severe acute respiratory syndrome coronavirus 2 (SARS-CoV-2) variant BA.2.86 detected in Denmark, including molecular characteristics and results from wastewater surveillance that indicate that the variant is circulating in the country at a low level. This new variant with many spike gene mutations was classified as a variant under monitoring by the World Health Organization on 17 August 2023. Further global monitoring of COVID-19, BA.2.86 and other SARS-CoV-2 variants is highly warranted.

Between 26 July and 21 August 2023, 10 human infections with severe acute respiratory syndrome coronavirus 2 (SARS-CoV-2) variant BA.2.86 were detected in Denmark. On 17 August, the World Health Organization (WHO) classified BA.2.86 as a variant under monitoring. The new variant, with a range of new spike (S) gene mutations, was quickly detected in several countries, including Israel, South Africa, the United Kingdom and the United States (US), suggesting international transmission. Here, we report on the first cases detected and surveillance data from Denmark, including epidemiological data and molecular characteristics of the variant.

## Description of the first cases

From 26 July to 21 August, surveillance detected 876 SARS-CoV-2 infections among 8,756 tested people from the 5,944,145 inhabitants of Denmark [1]. Of these, 418 were sequenced and 10 (2.4%) cases of the new variant BA.2.86 were detected. Variants EG.5.1 and XBB.1.16 were found among the majority of sequenced samples during this period (see the Supplement for a graphical distribution of the most prevalent variants).

Nine of the 10 cases were tested on clinical indication according to national guidelines [2]. The remaining case was detected by a surveillance project where individuals in several workplaces in Denmark can sign up to perform self-administered swabs at home when they have mild symptoms [3]. In total, 67 samples were tested as part of this surveillance project at workplaces from 26 July to 21 August.

Based on registry data, seven cases were female and three were male. Mean age was 57 years, median was 49 years, and five of the cases had at least one chronic condition. Seven of the cases lived in Greater Copenhagen region. All aged 18 years or above had been vaccinated against COVID-19 at least three times, with the latest vaccination 299–616 days before the BA.2.86 infection. Some of the cases had received the BA.1 updated vaccine as their fourth vaccination. Five of the cases had had a PCR-confirmed SARS-CoV-2 infection 506–556 days before the BA.2.86 infection.

Eight of the cases were available for interview by 31 August. Interviews with these revealed that some belonged to the same household, while epidemiological links and geographical clustering were not apparent for most, including the first cases detected. For most there was no recent relevant travel history. Symptoms reported were similar to those seen for other variants, including cough, shortness of breath and fever. Some had an underlying disease or received immune-modulating treatment. None were severely ill. 

## BA.2.86 can be detected with variant-PCR

Samples from four cases were available for variant-specific qPCR analysis. The samples contained the ΔH69/V70 mutation, as detected with a BA.2.-specific allelic discrimination assay based on deletion-specific TaqMan probes [4]. The variant could be detected with a novel purpose-designed dsDNA dye-based PCR. The presence of the BA.2.86-specific S gene insertion ins16_MPLF was identified in three samples using a dsDNA dye-based PCR with insertion-specific primers, which are listed in the Supplement. The ins16_MPLF could not be detected in one sample, probably due to low viral load (E gene qPCR quantification cycle Cq = 37). Furthermore, to discriminate between the BA.2.86 variant and the two most dominant variants circulating in Denmark, EG.5 and XBB.1, we used a drop-out PCR that targets the ΔV483 mutation not present in the BA.2.86 variant to confirm the ins16_MPLF assay. We provide the PCR protocol in the Supplement.

## Isolation of virus attempted in different cell lines

By 31 August 2023, virus isolation has been attempted without success from one case on Vero E6 cells and from three cases on Vero E6 cells and Vero E6 cells overexpressing angiotensin-converting enzyme 2 (ACE2) and TMPRSS2 [5].

## Whole genome sequencing and molecular clock

**Figure 1:**
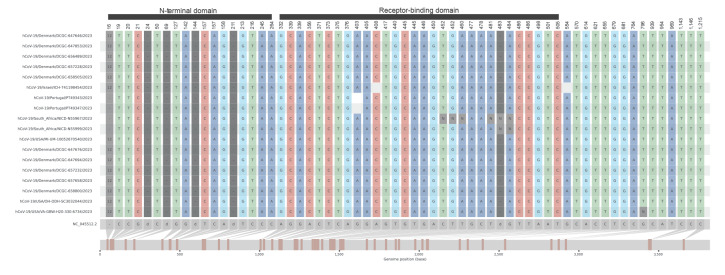
*snipit* plot showing nucleotide substitutions, insertions and deletions in SARS-CoV-2 BA.2.86 spike genes, Denmark until 28 August 2023 (n = 10) compared with sequences from global surveillance in the same period

We carried out amplicon-based whole genome sequencing using a modified ARTIC v3 primer scheme (github.com/artic-network/primer-schemes/tree/master/nCoV-2019/V3) or Arctic-midnight ONT V3, and consensus genomes were derived using a custom pipeline [6] or the pipeline wf-artic (v0.3.25) (https://github.com/epi2me-labs/wf-artic). We obtained lineage designations for each consensus genome using Nextclade (clades.nextstrain.org). Comparison of all Danish BA.2.86 genomes with others identified in global surveillance efforts from Israel, Portugal, South Africa and the US, showed 122 mutations with more than 50% conservation relative to the Wuhan-Hu1 reference genome, with a large proportion of these mutations (n = 79) in the S gene. These included a 12 nt insertion at S:16MPLF and four deletions in the S gene; the most interesting of these was S: ΔV483 in the receptor binding motif (RBM). We also observed the S:E484K mutation (also located in the RBM), which had not previously been observed in the Omicron lineage (Figure 1). We also observed several mutations in the N-terminal domain (NTD) antigenic supersite of the S gene, which suggests altered antigenicity of the S protein.

SARS-CoV-2: severe acute respiratory syndrome coronavirus 2.

Sequences were retrieved from GISAID. Contiguous insertions and deletions are merged to the first position in the range. All positions shown are amino acid positions relative to the Wuhan-Hu1 reference spike protein. Amino acid ranges corresponding to the N-terminal and receptor-binding domains are indicated with black lines.

**Figure 2:**
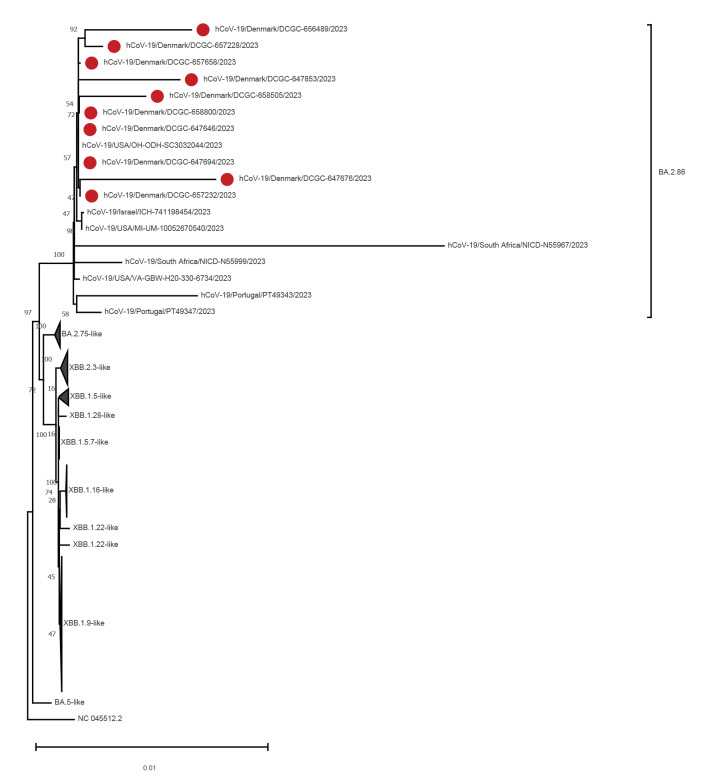
Maximum-likelihood phylogeny showing BA.2.86 genomes from Denmark (n = 10) and global GISAID submissions until 28 August 2023 as well as representative genomes from other SARS-CoV-2 lineages circulating in Denmark

We performed maximum-likelihood phylogenetic analysis with all 10 Danish genomes as well as eight genomes from global surveillance and representative genomes (at a redundancy threshold of 98%) of all lineages sequenced in Denmark over the sampling period of the first 10 cases. The BA.2.86 variant did not cluster with any circulating lineages and was distant from other known lineages circulating in Denmark (Figure 2). The BA.2.86 genomes fell into two distinct groups, and all genomes from Denmark grouped together with genomes from Israel and the US, suggesting parallel circulation in more than one location globally before the first samples were collected and sequenced.

SARS-CoV-2: severe acute respiratory syndrome coronavirus 2.

Included are sequences observed in Denmark between sampling of the first and most recent of the BA.2.86 cases. Sequences are grouped by nearest higher-order lineage. Representative genomes for each lineage group were derived using cd-hit (98% redundancy) and aligned to BA.2.86 genomes as well as the Wuhan-Hu1 reference genome (NC_045512.2) using the FFT-NS-2 algorithm in MAFFT [10]. Maximum likelihood trees were built with IQTREE2 [11], MODELFINDER [12] was used to obtain the most appropriate model (TN + F + G4). Shown is the consensus phylogeny from 1,000 bootstrap replicates, visualised in MEGA 11 [13]. Red node markers denote BA.2.86 genomes, other lineages with more than one genome per cluster are collapsed to black triangles.

Determination of the time to most recent common ancestor (TMRCA) for this lineage used a maximum-likelihood phylogeny of BA.2.86 genomes globally as input in BEAST (v.1.10.4) with an uncorrelated relaxed clock model, a coalescent exponential growth tree prior and Markov chain Monte Carlo chains run for 10,000,000 generations [7]. The TMRCA was estimated to be 15 June 2023 with a 95% HPD between 9 April to 24 July 2023.

## Wastewater surveillance

Surveillance of the new variant in wastewater was initiated after the first cases were detected. Samples from the Danish COVID-19 wastewater surveillance programme from 7 to 17 August 2023 were subjected to SARS-CoV-2 sequencing as described above (29 wastewater plants sampled twice per week, n = 116). Freyja v.1.4.5 (barcode version 08_22_2023–00–20) [8] was used to estimate lineage abundance in the samples, using 10 × as depth cutoff.

More than 50% coverage was obtained for 86 (74%) of the wastewater samples. The BA.2.86 variant was not detected by Freyja in any of the samples, but some contained multiple mutations characteristic of BA.2.86 (e.g. A7842G, C8293T and G8393A or C897A and C8293T). None of the 10 cases live in the catchment areas where the characteristic mutations were found.

The first results from wastewater surveillance of the following week (sampled on 22 and 24 August 2023) showed that characteristic mutations are continuously identified in a number of samples, and BA.2.86 was detected by Freyja at 3.6% abundance at one location.

## Discussion

The SARS-CoV-2 BA.2.86 variant causes concern because a large number of mutations distinguish it from previous variants. While the limited epidemiological data available indicate that the variant is already dispersed across Denmark, the proportion of BA.2.86 among sequenced samples has been small and cannot explain the recently observed increasing transmission (see the figure about increase in the Supplement). Further, none of the 10 BA.2.86 cases have been severely ill. It is, however, crucial to underline that this is a preliminary assessment based on the first cases.

When a new variant of a pandemic pathogen emerges, quick initial assessment and sharing of information internationally is crucial. Related strengths in Denmark include having both diagnostic and epidemiological preparedness for infectious diseases located at the same institute, Statens Serum Institut, and a close collaboration with the regional departments of clinical microbiology that perform diagnostic tests, sequencing and initial bioinformatic analyses.

Denmark retains active genomic surveillance for emerging variants, but testing activity now is much lower and the coverage of the national testing policy more limited than it was at other time points during the pandemic, which affects the sensitivity to detect new variants and introduces referral bias. Our results showcase the usefulness of complementary surveillance systems. Wastewater surveillance allowed us to assess the general level of circulation and spread across the country [9], and the surveillance project at workplaces contributed with samples from individuals with mild symptoms.

Importantly, continuous monitoring of SARS-CoV-2 infections and other indicators as well as the distribution of variants signalled the emergence of BA.2.86 and that it thus far had not substantially contributed to the overall increase in infections. Close monitoring of the variant continues, with a focus on its growth advantage, clinical severity and possible immune-evasive properties.

## Conclusions

Given the widespread distribution of BA.2.86 globally and within Denmark in clinical samples and in wastewater, and the high number of mutations conferring immune-evasive potential, the possibility that BA.2.86 will outcompete currently dominating variants cannot be ruled out. Our very early clinical data suggest a clinical picture in line with the typical COVID-19 illness caused by previous variants. Data are presently very preliminary and further global monitoring of BA.2.86 and other SARS-CoV-2 variants is highly warranted.

## Supplementary Data

1538000 Supplement
