# Supplementary material for: First cases of SARS-CoV-2 BA.2.86 in Denmark, 2023
Source: Euro Surveill. 2023 Sep 7;28(36):2300460. doi: 10.2807/1560-7917.ES.2023.28.36.2300460 (PMC10486197; doi:10.2807/1560-7917.ES.2023.28.36.2300460)
Supplement: Supplement [file 23-00460_JOKELAINEN_Supplement.pdf]

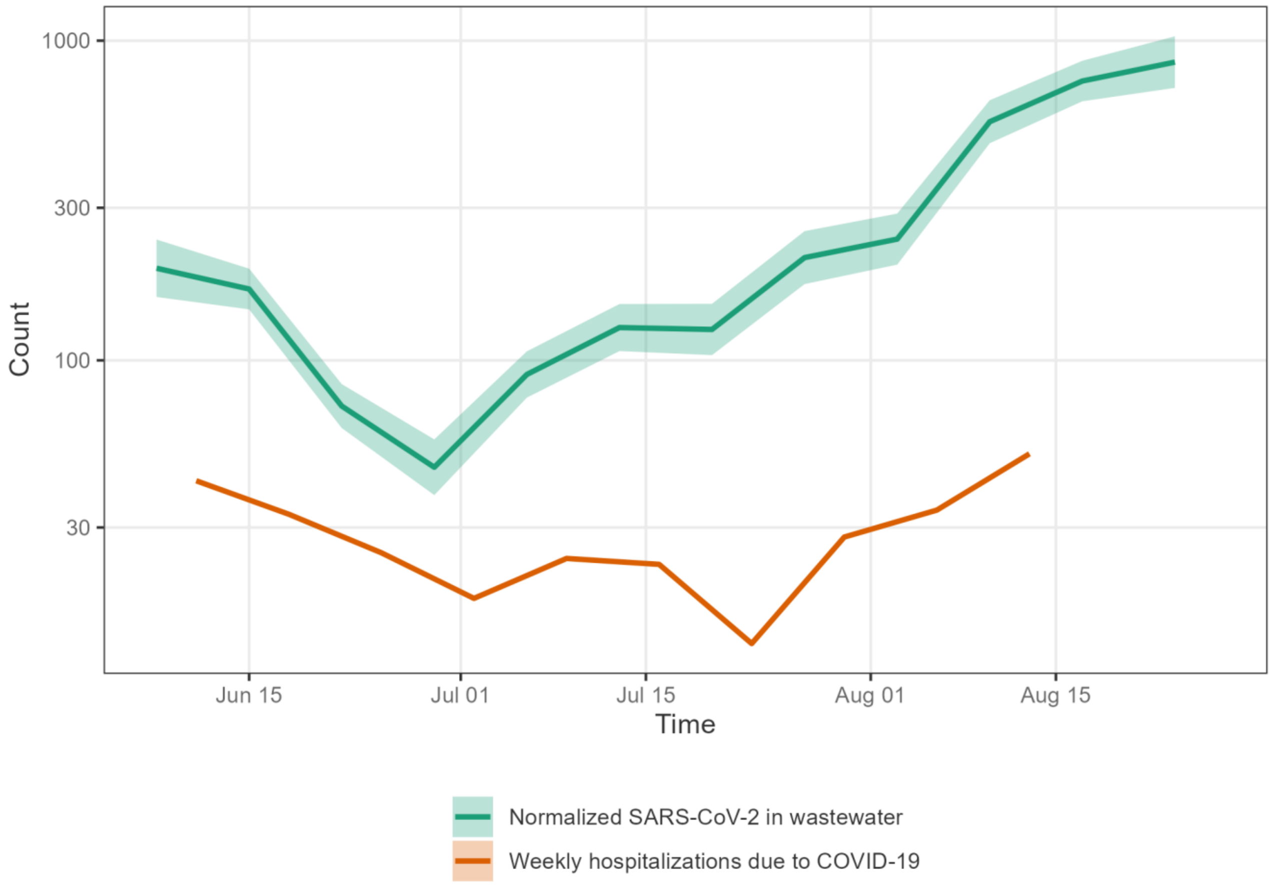

Supplementary Material
The weekly number of hospitalizations due to COVID-19 in Denmark and the normalized national average concentration of SARS-CoV-2 in Denmark from 4 June 2023 to 27 August 2023. The concentration in the wastewater has grown exponentially since the beginning of July resulting in a factor 20 increase over a period of eight weeks. The rapid growth precedes the known occurrences of the new BA.2.86 variant and the low abundance among sequenced cases underlines that the observed growth is due to other variants. Preliminary analysis using logistic regression shows a positive but not significant growth in the proportion of sequenced cases. In combination with the growth seen in the wastewater then this indicates that the BA.2.86 is most likely growing in incidence.

This supplementary material is hosted by Eurosurveillance as supporting information alongside the article 'First cases of SARS-CoV-2 BA.2.86 in Denmark, 2023', on behalf of the authors, who remain responsible for the accuracy and appropriateness of the content. The same standards for ethics, copyright, attributions and permissions as for the article apply. Supplements are not edited by Eurosurveillance and the journal is not responsible for the maintenance of any links or email addresses provided therein.

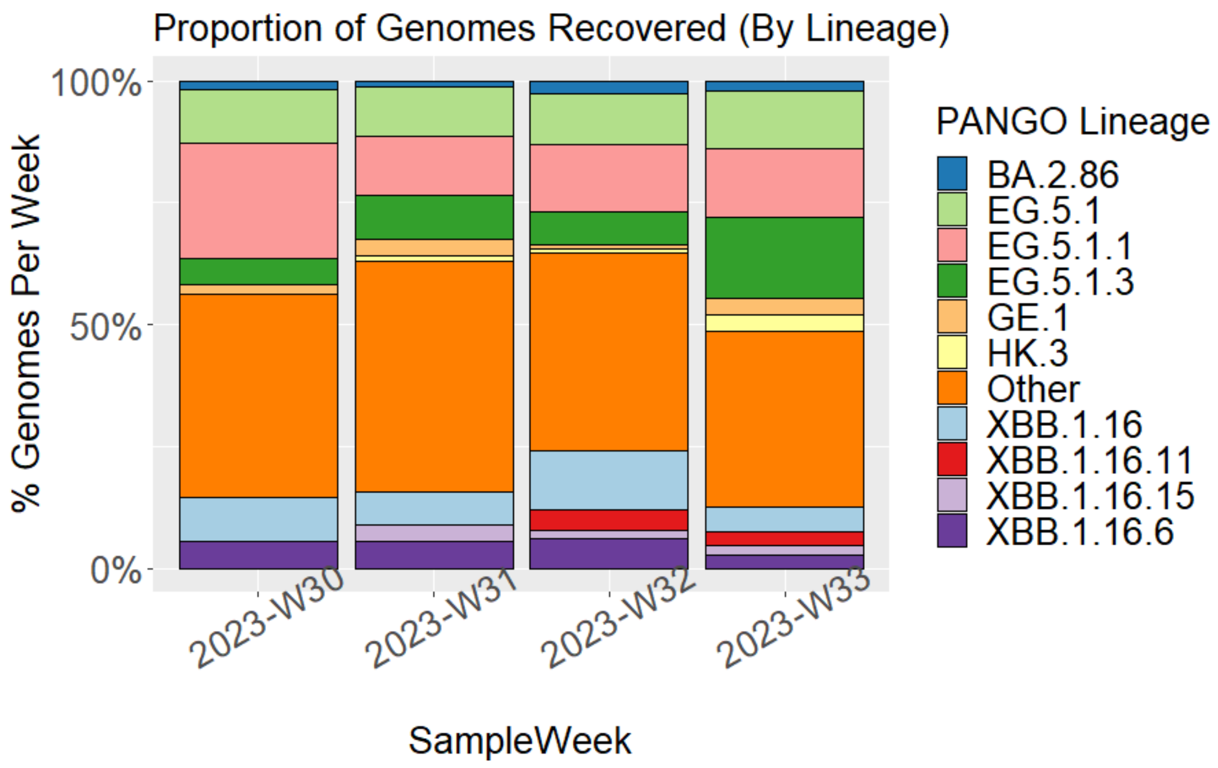

Supplementary material: Proportion of top 10 most prevalent SARS-CoV-2 Pango lineages recovered by whole genome sequencing in Denmark for the study period between week 30 – week 33, 2023.

This supplementary material is hosted by Eurosurveillance as supporting information alongside the article 'First cases of SARS-CoV-2 BA.2.86 in Denmark, 2023', on behalf of the authors, who remain responsible for the accuracy and appropriateness of the content. The same standards for ethics, copyright, attributions and permissions as for the article apply. Supplements are not edited by Eurosurveillance and the journal is not responsible for the maintenance of any links or email addresses provided therein.

DNA-binding dye-based BA.2.86 variant PCR assays

Primers:

Name	Sequence
delV483_Fw	TGGACCTTGAAGGAAAAGAGGG
delV483_Rv	CTGAGGGAGATCACGCTCTAAAT
ins16_MPLF_Fw	GTCATGCCGCTGTTTAATCTTATAAC
ins16_MPLF_Rv	TTGTCAGGGTAATAAACACCACG

Final PCR reaction mix for both assays:

Reagent	Final concentration
Luna ProbeOne-Step RT-qPCR (NEB)	1x
Luna WarmStart-RT (NEB)	1x
Fwd primer	250nM
Rv primer	250nM
EvaGreen DNA dye	1µM

PCR protocol:

Step	Temperature	Time	Cycles
Reverse transcription	55°C	10 min	1x
Polymerase activation	95°C	3 min	1x
Denaturing	95°C	10 sec	40x
Annealing	60°C	30 sec	40x

Criteria for BA.2.86 call:

BA.2.86 variant if: ins16_MPLF assay Ct < 38 AND no Ct in delV483 assay

BA.2.86 variant if: ins16_MPLF assay Ct 10 or more cycles lower than Ct of delV483 assay

This supplementary material is hosted by Eurosurveillance as supporting information alongside the article 'First cases of SARS-CoV-2 BA.2.86 in Denmark, 2023', on behalf of the authors, who remain responsible for the accuracy and appropriateness of the content. The same standards for ethics, copyright, attributions and permissions as for the article apply. Supplements are not edited by Eurosurveillance and the journal is not responsible for the maintenance of any links or email addresses provided therein.

SUPPLEMENTAL TABLE

Data Availability

GISAID Identifier:	EPI_SET_230830yq
doi:	10.55876/gis8.230830yq

All genome sequences and associated metadata in this dataset are published in GISAID's EpiCoV database. To view the contributors of each individual sequence with details such as accession number, Virus name, Collection date, Originating Lab and Submitting Lab and the list of Authors, visit 10.55876/gis8.230830yq

Data Snapshot

- EPI_SET_230830yq is composed of 8 individual genome sequences.
- The collection dates range from 2023-07-24 to 2023-08-15;
- Data were collected in 4 countries and territories;
- All sequences in this dataset are compared relative to hCoV-19/Wuhan/WIV04/2019 (WIV04), the official reference sequence employed by GISAID (EPI_ISL_402124). Learn more at https://gisaid.org/WIV04.

This supplementary material is hosted by Eurosurveillance as supporting information alongside the article 'First cases of SARS-CoV-2 BA.2.86 in Denmark, 2023', on behalf of the authors, who remain responsible for the accuracy and appropriateness of the content. The same standards for ethics, copyright, attributions and permissions as for the article apply. Supplements are not edited by Eurosurveillance and the journal is not responsible for the maintenance of any links or email addresses provided therein.
